# The RAST Server: Rapid Annotations using Subsystems Technology

**DOI:** 10.1186/1471-2164-9-75

**Published:** 2008-02-08

**Authors:** Ramy K Aziz, Daniela Bartels, Aaron A Best, Matthew DeJongh, Terrence Disz, Robert A Edwards, Kevin Formsma, Svetlana Gerdes, Elizabeth M Glass, Michael Kubal, Folker Meyer, Gary J Olsen, Robert Olson, Andrei L Osterman, Ross A Overbeek, Leslie K McNeil, Daniel Paarmann, Tobias Paczian, Bruce Parrello, Gordon D Pusch, Claudia Reich, Rick Stevens, Olga Vassieva, Veronika Vonstein, Andreas Wilke, Olga Zagnitko

**Affiliations:** 1 Fellowship for Interpretation of Genomes, Burr Ridge, IL 60527, USA; 2 Mathematics and Computer Science Division, Argonne National Laboratory, Argonne, IL 60439, USA; 3 Computation Institute, University of Chicago, Chicago, IL 60637, USA; 4 Department of Microbiology, University of Illinois at Urbana-Champaign, Urbana, IL 61801, USA; 5 The Burnham Institute, San Diego, CA 92037, USA; 6 National Center for Supercomputing Applications, University of Illinois at Urbana-Champaign, Urbana, IL 61801, USA; 7 Hope College, Holland, MI 49423, USA; 8 University of Tennessee, Health Science Center, Memphis, TN 38136, USA; 9 Department of Microbiology and Immunology, Cairo University, Cairo, Egypt

## Abstract

**Background:**

The number of prokaryotic genome sequences becoming available is growing steadily and is growing faster than our ability to accurately annotate them.

**Description:**

We describe a fully automated service for annotating bacterial and archaeal genomes. The service identifies protein-encoding, rRNA and tRNA genes, assigns functions to the genes, predicts which subsystems are represented in the genome, uses this information to reconstruct the metabolic network and makes the output easily downloadable for the user. In addition, the annotated genome can be browsed in an environment that supports comparative analysis with the annotated genomes maintained in the SEED environment.

The service normally makes the annotated genome available within 12–24 hours of submission, but ultimately the quality of such a service will be judged in terms of accuracy, consistency, and completeness of the produced annotations. We summarize our attempts to address these issues and discuss plans for incrementally enhancing the service.

**Conclusion:**

By providing accurate, rapid annotation freely to the community we have created an important community resource. The service has now been utilized by over 120 external users annotating over 350 distinct genomes.

## Background

In 1995 the first complete genome became available. Since then, hundreds more have been sequenced, and it has become clear that thousands will follow shortly. This has led to the obvious conclusion that most of the annotations that will be associated with these newly-sequenced genomes will be provided through technologies that are largely automated, and a growing number of efforts focusing on different aspects of automated annotation have emerged [1-6]. In this paper we describe the RAST Server, a fully automated annotation service for complete, or near-complete, archaeal and bacterial genomes. The service seeks to rapidly produce high-quality assessments of gene functions and an initial metabolic reconstruction. Initially the server was planned for use by the National Microbial Pathogen Data Resource (NMPDR) [7] community, but very quickly the global utility of such a service became apparent. Users of the facility upload a genome as a set of contigs in FASTA format, and they receive access to an annotated genome in an environment that supports comparison with an integration of hundreds of existing genomes. The complete annotation is normally produced within 12–24 hours, and the existing implementation can support a throughput of 50–100 genomes per day. However, it is important to note that speed is not the central requirement for such a system; accuracy, completeness and consistency will ultimately be the criteria used to evaluate the success or failure of a service such as the one described. To date, the server has been used by over 120 external users to annotate over 350 genomes.

RAST bases its attempts to achieve accuracy, consistency, and completeness on the use of a growing library of *subsystems *that are manually curated [8], and on protein families largely derived from the subsystems (*FIGfams*). In the sections below we describe the steps the RAST server implements to automatically produce two classes of asserted gene functions: *subsystem-based assertions *are based on recognition of functional variants of subsystems, while *nonsubsystem-based assertions *are filled in using more common approaches based on integration of evidence from a number of tools. The fact that RAST distinguishes these two classes of annotation and uses the relatively reliable subsystem-based assertions as the basis for a detailed metabolic reconstruction makes the RAST annotations an exceptionally good starting point for a more comprehensive annotation effort.

Besides producing initial assignments of gene function and a metabolic reconstruction, the RAST server provides an environment for browsing the annotated genome and comparing it to the hundreds of genomes maintained within the SEED [9] integration. The genome viewer included in RAST supports detailed comparison against existing genomes, determination of genes that the genome has in common with specific sets of genomes (or, genes that distinguish the genome from those in a set of existing genomes), the ability to display genomic context around specific genes, and the ability to download relevant information and annotations as desired.

## Construction and content

### Subsystems: an Overview

It is commonly held that one central role of bioinformatics is to project a relatively small set of assertions of gene and protein function from the literature (i.e., from wet lab characterizations) to genes from other genomes. This captures a kernel of truth (that, ultimately, new assertions of function are based on wet lab characterizations), but, perhaps, elevates the role of bioinformatics beyond what is reasonable to expect. In contrast, we view projection as a 2-step process:

1. In an initial stage, an expert in a biological topic integrates what is known from the literature producing a set of *expert assertions*, which include the assertions from the literature, as well as a far broader set based on judgement and extrapolation.

2. Bioinformatics tools are developed to project structured collections of expert assertions (rather than just the wet lab results captured in the literature) to new genomes.

The process of integrating what is known from the literature into a set of expert assertions involves highly complex decisions and is well beyond most of the common bioinformatics tools. On the other hand, there is every reason to believe that fully automated tools can be developed to project these expert assertions. The more comprehensive and well structured the collection of expert assertions, the more rapidly accurate projection technology will be developed. Here it is worth noting that we speak of "well-structured" sets of expert assertions, since the developed tools will almost certainly need to encapsulate numerous rules covering special cases, and a careful delineation of these rules can best be achieved by domain experts.

One technology for creating and maintaining expert assertions was developed within the context of *The Project to Annotate 1000 Genomes *[10].

**Figure 1:**
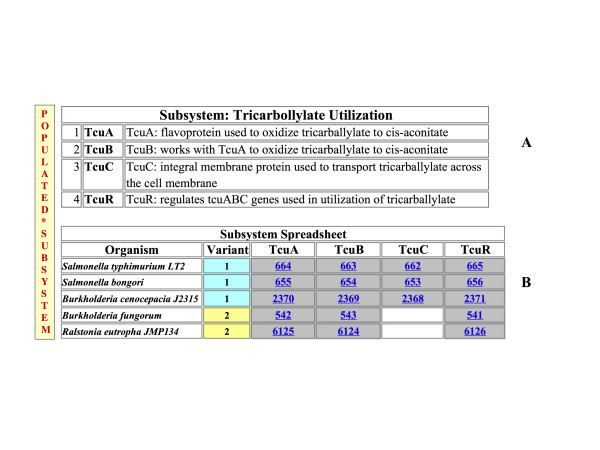
**Example Tricarballylate Utilization Subsystem**. A) The subsystem is comprised of 4 functional roles. B) The Subsystem Spreadsheet is populated with genes from 5 organisms (simplified from the original subsystem) where each row represents one organism and each column one functional role. Genes performing the specific functional role in the respective organism populate the respective cell. Gray shading of cells indicates proximity of the respective genes on the chromosomes. There are two distinct variants of the subsystem: variant 1, with all 4 functional roles and variant 2 where the 3rd functional role is missing.

This technology involves an expert curator defining a *subsystem *as a set of abstract *functional roles*. Figure 1A shows a very simple case in which a subsystem named "Tricarballylate Utilization" is composed of four functional roles. The subsystem is *populated *by connecting these functional roles to specific genes in specific genomes, producing a *subsystem spreadsheet*, where each row represents one genome and each column corresponds to one functional role as shown in Figure 1B. The proteins encoded by the genes in one column are used to construct the subsystem-based FIGfams (discussed below). The cooperative effort to develop subsystems has produced a publicly available set of such populated subsystems that now includes over 600 subsystems. These subsystems include assertions of function for well over 500,000 protein-encoding genes in over 500 bacterial and archaeal genomes (relating to over 6200 functional roles). This manually curated collection represents sets of co-curated protein families. While it is true that the quality of the assertions varies substantially, it is also true that these structured sets of assertions represent a major resource in constructing automated annotation systems.

### FIGfams: Yet Another Set of Protein Families

A number of groups have spent substantial effort building protein families that now represent resources that are widely used and valued by the community [11-15]; see [16] for a more extended discussion. RAST utilizes a new collection of protein families. This collection is referred to as the set of *FIGfams*, and the publication of a detailed account of them is in preparation. Each FIGfam may be thought of as a 3-tuple composed of a set of proteins, a family function, and a decision procedure. The set of proteins are believed to be globally similar (and, presumably, homologous) and the members all share a common function. The decision procedure takes as input a protein sequence and returns a decision about whether or not the protein could be added to the family (i.e., whether or not the protein is globally similar to the members and shares the common function).

Hence, the basic principles underlying FIGfams are quite similar to those corresponding to the lowest-level PIR families [17] or the TIGRfam *equivalogs *[15].

The construction of FIGfams is done conservatively: care is taken to make sure that two proteins included in the same set actually do share a common function, but if substantial uncertainty exists about whether or not two proteins actually share the same function they are kept in distinct families. Two proteins will be placed in the same family:

1. If both occur in the same column of a manually curated subsystem spreadsheet (i.e., if they implement the same functional role) and the region of similarity shared by the two sequences covers over 70% of each sequence.

2. If they come from closely related genomes (e.g., genomes from two strains of the same species), the similarity is high (usually greater than 90% identity), and the context on the chromosome (i.e., the adjacent genes) can easily be seen to correspond, then they can be placed in the same family (even if the function they implement is yet to be determined).

These are the two cases in which we feel confident in asserting a common function between two proteins; the first reflects an expert assertion, and the second an instance in which divergence is minimal. Construction of FIGfams using these two grouping principles has led to a collection of about 17,000 FIGfams that include proteins related to subsystems (those are the FIGfams that we call subsystem-based) and over 80,000 that contain only proteins grouped using the second principle (i.e. the non-subsystem-based FIGfams). Many of the non-subsystem-based FIGfams contain just 2, 3 or 4 proteins.

Over time we expect to coalesce the non-subsystem-based FIGfams. This will be done by creating new, manually curated subsystems; these will form kernels of new families that will group the isolated families that now exist.

It is worth noting that the existing collection of FIGfams covers most of the central cellular machinery with families derived from subsystems, and the numerous small non-subsystem-based FIGfams efficiently support recognition of genes in close strains. While it is true that we cover a limited percentage of genes in newly sequenced divergent genomes, we recognize well over 90% of the genes in newly sequenced strains that are close to existing annotated genomes. It seems likely that a large percentage of newly sequenced genomes will be close to existing genomes (e.g., note projects to sequence tens and soon hundreds of closely related pathogenic strains), and the FIGfams already constitute an effective recognition framework in such cases.

### The Basic Steps in Annotating a Genome Using RAST

The basic steps used to annotate a genome using RAST are described in the subsections below. Input to the process is a prokaryotic genome in the form of a set of contigs in FASTA format. As described below, the actual RAST server will allow a user to specify a set of gene calls, but in the usual case RAST will make its own calls. We now describe the basic steps in a RAST annotation in detail.

### Call the tRNA and rRNA genes

We use existing tools built by other research teams to first identify both the tRNA and rRNA encoding genes. For the tRNA genes we use tRNAscan-SE [18] and to identify the rRNA encoding genes we use a tool " search_for_rnas" developed by Niels Larsen (available from the author). We begin the process by calling these genes, which we believe can be reliably determined. Then, the server will not consider retaining any protein-encoding gene that significantly overlaps any of these regions. Unfortunately, the public archives do contain putative protein-encoding genes that are embedded in rRNAs. These gene calls are almost certainly artefacts of the period in which groups were learning how to develop proper annotations, and RAST attempts to avoid propagating these errors.

### Make an Initial Effort to Call Protein-Encoding Genes

Once the tRNA and rRNA gene-encoding regions are removed from consideration, we make an initial call using GLIMMER2 [19]. At this point we are seeking a reasonable estimate of probable genes, and GLIMMER2 is an excellent tool for that purpose. At this stage, RAST is not concerned about calling spurious genes or getting starts called accurately. What is needed is that most of the actual protein-encoding genes are represented in the initial estimate of *putative genes*.

### Establishing Phylogenetic Context

Once an initial set of protein-encoding genes has been established, we take representative sequences from a small set of FIGfams that have the property that they are universal or nearly universal in prokaryotes. This set includes, for example, the tRNA synthetases.

Using this small set of representatives we search the protein-encoding genes from the new genome for occurrences of these FIGfams. It should be noted that this is a very rapid step, since only the new genome is being searched, and it is being searched using a small set of representative protein sequences. The outcome of this initial scan is a small set (normally, 8–15 genes) that can be used to estimate the closest phylogenetic neighbours of the newly-sequenced genome. This can be done by taking each located gene and blasting it against the genes from the corresponding FIGfam. Normally, we attempt to locate the ten closest neighbours, but clearly the approach is insensitive to the exact number sought. For each detected gene, we adjust its starting position and move it from the set of *putative genes *to a set of *determined genes *and the function (i.e., product name) assigned to the gene is taken from the FIGfam.

### A Targeted Search Based on FIGfams that Occur in Closely Related Genomes

Once the "neighbouring genomes" have been determined, we can form the set of FIGfams that are present in these genomes. This constitutes a set of FIGfams that are likely to be found in the new genome. For each of these FIGfams, we search the new genome. Note that we expect these searches to have a relatively high rate of success. Whenever we do find a gene, we adjust its starting position and move the gene from the set of *putative genes *to the set of *determined genes*. The computational costs required to locate these genes are low (since we are searching a very small set of putative genes).

### Recall Protein-Encoding Genes

At this point, we have accumulated a set of *determined genes *within the new genome and can now use this excellent training set to recall the protein-encoding genes. In the case of a genome that is a closely related strain of one or more existing genomes, this training set may well include over 90% of the actual protein-encoding genes.

### Processing the Remaining Genes Against the Entire FIGfam Collection

The *putative genes *that remain can be used to search against the entire collection of FIGfams. This is done by blasting against a representative set of sequences from the FIGfams to determine potential families that need to be checked, and then checking against each family. While computationally more expensive than the focused searches in the previous steps, it is still far, far cheaper than blasting against a large non-redundant protein database. Currently, the collection of representative protein sequences from FIGfams used to compute potentially relevant FIGfams includes somewhat over 100,000 protein sequences.

This step amounts to a comprehensive search of the FIGfams for each of the remaining putative genes. Once it has been completed, all of the genes that could be processed using FIGfams have been processed.

### Clean Up Remaining Gene Calls (Remove Overlaps and Adjust Starting Positions)

The *putative proteins *that remain are processed to attempt to resolve issues relating to overlapping gene calls, starts that need to be adjusted, and so forth. In the case of the RAST server, we do blast the remaining putative genes against a large non-redundant protein database in an attempt to determine whether there is similarity-based evidence that could be used in resolving conflicts.

### Process the Remaining, Unannotated Protein-encoding Genes

At this point, final assignments of function are made to the remaining putative genes. If similarities were computed in the preceding step, these similarities can be accessed and functions can be asserted. Optionally, one can employ any of the commonly employed pipeline technologies to run a suite of tools and produce a more accurate estimate. The genes processed using this approach represent most of the overhead in a RAST annotation. By first processing a majority of the genes using FIGfam-based technology and focused searches, this cost is minimized by RAST without (we believe) reducing accuracy.

### Construct an Initial Metabolic Reconstruction

**Figure 2:**
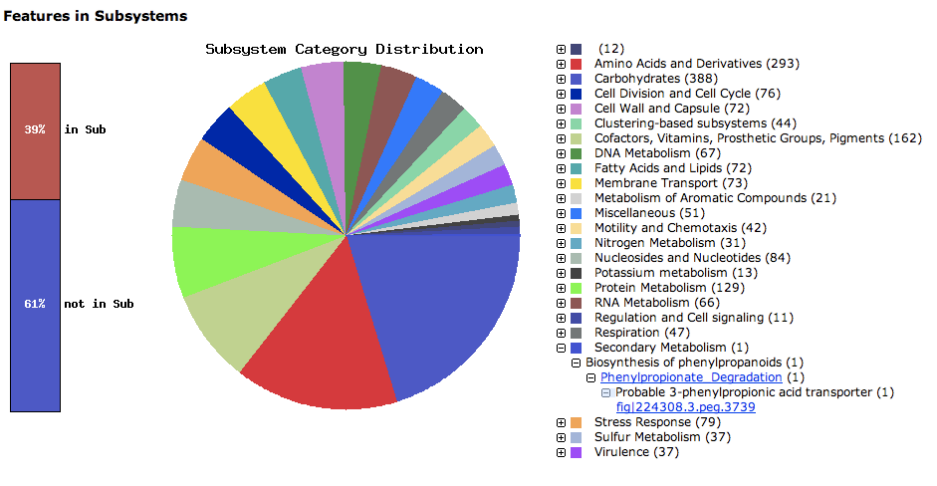
**Genes connected to subsystems and their distribution in different categories**. The categories are expandable down to the specific gene (see Secondary Metabolism).

Once assignments of function have been made, an initial metabolic reconstruction is formed. For our purposes, this amounts to connecting genes in the new genome to functional roles in subsystems, determining when a set of connections to a specific subsystem are sufficient to support an *active variant *of the subsystem, and tabulating the complete set of active variants. Since the subsystems themselves are arranged in crude categories reflecting basic divisions of function, we can produce a detailed estimate of the genome contents that got successfully connected to subsystems (see Figure 2). In the case of a genome like *Buchnera aphidicula*, in excess of 82% of the genes fall in this category; for *Escherichia coli *O157:H7 the percentage drops to 76%, while in a relatively diverged genome like *Methanocaldococcus jannaschii *DSM 2661 the percentage that can be connected (at this point in time) is only 22%. Figure 2 offers a brief overview of the type of display a user can employ to quickly explore the contents of the new genome.

It should be emphasized that the subsystems cover all modules of cellular machinery – not just the metabolic pathways. Hence, what we are calling a *metabolic reconstruction *(a collection of the active variants of subsystems that have been identified) is more properly thought of as a grouping of genes into modules, rather than the reconstruction of the metabolic network. However, besides simply compiling the set of active variants of subsystems, the RAST server uses a set of *scenarios *encoded in metabolic subsystems to assemble a metabolic reaction network for the organism [20]. These scenarios represent components of the metabolic network in which specific compounds are labelled as inputs and outputs (i.e., they may be thought of as directed modules of the metabolic network). The metabolic network is assembled using biochemical reaction information associated with functional roles in subsystems to find paths through scenarios from inputs to outputs. Scenarios that are connected by linked inputs and outputs can be composed to form larger blocks of the metabolic network, spanning processes that convert transported nutrients into biomass components. In the case of newly sequenced genomes that are close to those our team manually curates, it is possible to directly estimate what percent of the reaction network typically included in a genome-scale metabolic reconstruction [21] can be generated automatically. Today the RAST server produces 70–95% of the reaction network, depending on the specific species and genome.

## Utility

In the previous sections we have described the basic technology that underlies the RAST server. We believe that the issues discussed above determine accuracy and speed of the system. The usability of the system is largely determined by the user interface.

We have spent the effort required to build a simple interface that offers the ability to submit genomes, monitor progress of the annotation, to view the results in a framework allowing comparisons against hundreds of existing genomes, and the ability to download the results in any of several formats.

### Upload Genome and monitor annotation process

The service is freely available for the annotation of prokaryotic genomes. The genomes may be "complete" or they may be in hundreds of contigs (which does impact the quality of the derived annotations). A new user must *register *for the service, which involves giving us contact information and acquiring a password. By registering users, we can create a framework in which users have access to only those genomes that they have submitted. It allows us also to contact the user once the automatic annotation has finished or in case user intervention is required.

**Figure 3:**
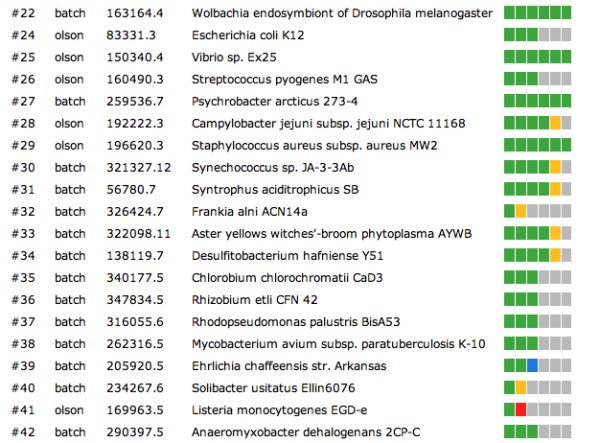
**Job Overview page**. The colours in the progress bar have the following meaning: gray – not started, blue – queued for computation, yellow – in progress, red – requires user input, brown – failed with an error, green – successfully completed.

**Figure 4:**
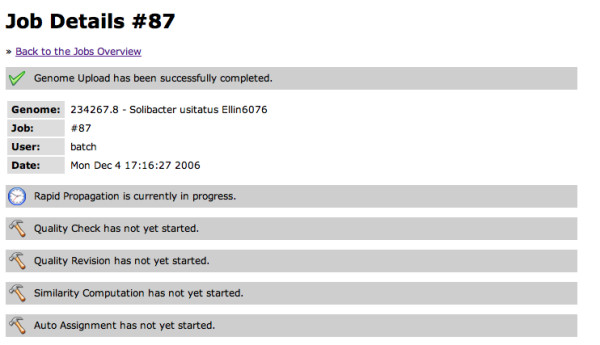
**Job Detail page**. The RAST annotation progress can be monitored by each user.

After login the user can monitor his/her submitted job/jobs on the Job Overview page (Figure 3). This page lists for each submitted job its number, submitter, the taxonomy ID and Genome name followed by a six-button bar, where each button represents a step in the RAST annotation service. Depending on the state of each step the button colour will change from grey (not started) to blue (queued for computation) to yellow (in progress) to green (successfully completed) or red (error) as shown in Figure 3. More detailed information about each step can be viewed after clicking the button bar itself. Figure 4 illustrates such a Job Detail page with the submission time stamp and the six steps. Here step one had been completed, step two was in progress and the other steps had not yet started.

### Browse Genome in SEED-Viewer environment

After the annotation is complete the user can choose to download the annotated genome in a variety of export formats (e.g. GenBank, FASTA, GFF3, Excel) or browse the genome in the comparative environment of the SEED-Viewer without having the data actually installed in the SEED. These options remain for 120 days or until the data are deleted by the user. If desired, the user can request to have the annotated genome added to the SEED.

The SEED-Viewer environment presents the user with a variety of options for the immediate analysis of the annotated genome. The Organism Overview page contains basic information on the Genome such as Taxonomy, Size, the Number of Contigs, the Number of Coding Sequences and RNAs and counts of non-hypothetical and hypothetical gene annotations. In addition it contains the Number of Subsystems that were automatically determined to be present in the genome. A bar graph and a pie chart (shown in Figure 2) illustrate the distribution of genes connected to the various subsystem groups. Each of those groups can be expanded (by clicking the "+" button) down to the specific protein encoding genes (pegs) found in a given subsystem. This page is also the entry point to a whole Genome Browser, the Compare Metabolic Reconstruction tool, the View Features and the View Scenarios pages.

**Figure 5:**
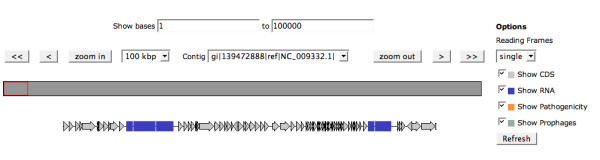
**Genome Browser**. The annotated genome can be browsed starting from a whole-genome view and zooming-in to a specific feature.

**Figure 6:**
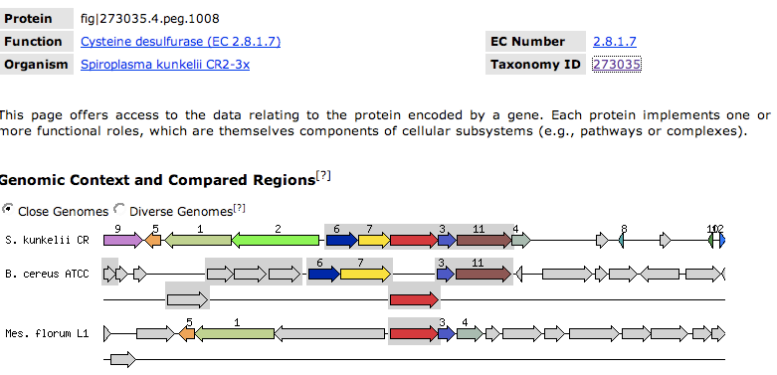
**Annotation Overview**. For each annotated feature RAST presents an overview page, which includes comparative genomics views and the connections to a subsystem if one was asserted.

The whole Genome Browser, as shown in Figure 5 allows the user to zoom from a graphic whole genome presentation into any desired area of the genome down to a gene (peg or RNA encoding gene). By clicking at any of the genome features the user can choose to see the Annotation Overview page (Figure 6), which includes a graphical representation of the Genomic Context of the peg of interest and compares that to regions in other genomes that have homologous genes.

**Figure 7:**
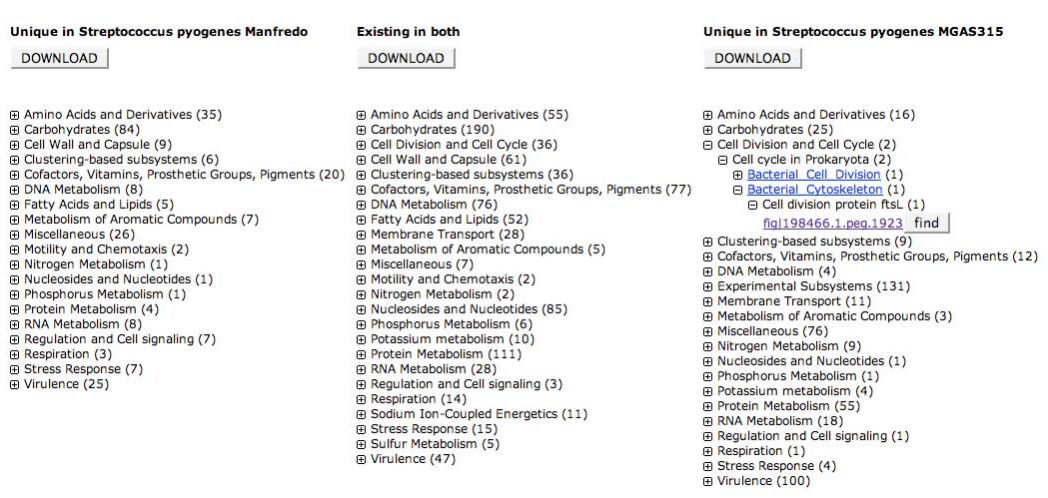
**Compare Metabolic Reconstruction tool**. In the example the RAST metabolic reconstruction of the submitted genome of *S. pyogenes *Manfredo was compared to the metabolic reconstruction for *S. pyogenes *MGAS315, which is part of the comparative environment of the SEED. All three columns of subsystem categories are expandable. In cases where RAST was conservative in the assertion of a subsystem a manual attempt to retrieve the missing function/s can be made by clicking the find button.

The Compare Metabolic Reconstruction tool allows the user to compute a metabolic comparison of the newly-annotated genome to any genome present in the SEED. The output of such a computation is a three-column table (Figure 7) that shows genes that are connected to subsystems and are unique in the query genome (left column) or unique in the SEED genome (right column) or are found in both genomes (middle column). To see individual genes the user needs to un-collapse the three-tear hierarchical representation of subsystems (by clicking the "+" buttons).

**Figure 8:**
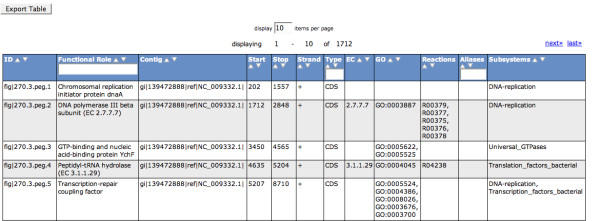
**View Features page**. All annotated features can be viewed and downloaded in table format. For each peg the location on the contig, the functional role assignment, its EC number (if present) and GO category, the connection to a subsystem and a KEGG reaction (if appropriate) are given.

All annotated features can be viewed and downloaded from the View Features page (Figure 8). For each peg the location on the contig, the functional role assignment, its EC number (if present) and GO category, the connection to a subsystem and a KEGG reaction (if appropriate) are displayed.

**Figure 9:**
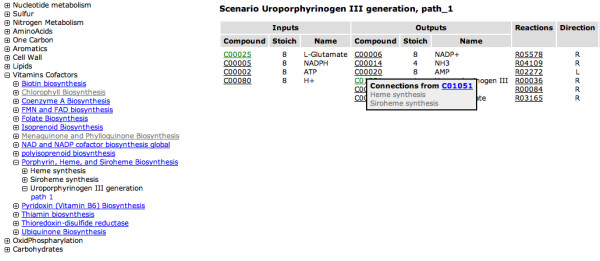
**View Scenarios page**. A genome-specific reaction network can be viewed on a scenario by scenario basis. The scenarios are organized on the left by subsystems, which are themselves organized by categories of metabolic function. If a path through a scenario was found in a given subsystem, the subsystem name is highlighted in blue. In this case, one path was found through the Uroporphyrinogen III generation scenario in the Porphyrin, Heme and Siroheme Biosynthesis subsystem. The table to the right shows the input and output compounds for the scenario, including their stoichiometry, and the reactions that make up the path through the scenario.

For each annotated genome a set of metabolic scenarios is computed and can be viewed on the View Scenarios page (Figure 9). Again a subsystem hierarchy can be un-collapsed and for each subsystem that has been asserted, a scenario is given with input and output compounds, their stoicheometry and a relevant coloured KEGG map (if one exists).

## Discussion

A beta version of the RAST server was made available in February 2007. Since then we have been addressing performance issues, systematic errors, and all of the details required to effectively support such a service. Over 120 external users have now registered, and we have processed over 350 submissions from these users. The total number of genomes processed exceeds 1200 (including genomes that we have run through the system for evaluation purposes and to recall annotations in some of the existing genomes) at the time of writing this manuscript.

### Performance analysis

**Figure 10:**
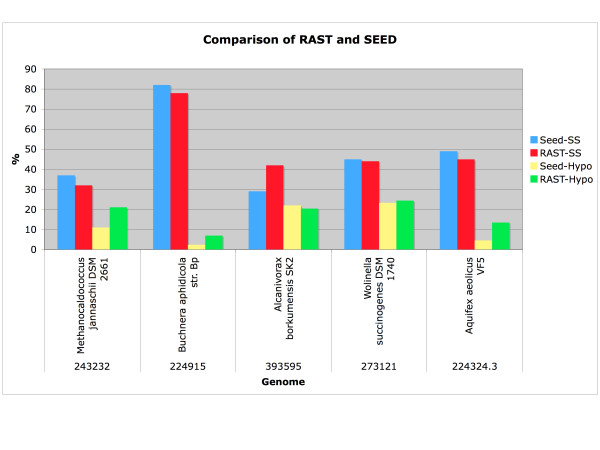
**Comparison of a set of genomes manually curated in the SEED and automatically annotated in RAST**. The number of genes annotated as hypothetical and the number of genes linked to subsystems (our mechanism of manual curation) is shown to provide an initial assessment of the performance of RAST.

To provide an assessment of the annotation quality of the new service, we first have compared the annotations in our manually curated SEED annotation framework with those generated automatically by the RAST server. There are obvious limitations in using existing SEED genomes to evaluate the service, and this lead us to add a comparison of RAST annotations to KAAS (KEGG Automatic Annotation Server) [22] annotations, the only other public annotation service that we are aware of which will allow an online sequence submission. The output of this comparison is available online, please see the section on availability. A rough estimate of annotation quality can be gained by comparing the number of genes linked to subsystems [8] and the number of genes annotated as hypothetical proteins (see Figure 10).

This informal analysis indicates that the RAST server can successfully project the annotations generated in the SEED environment, as the number of hypotheticals and the number of genes linked to subsystems are roughly equivalent for RAST and SEED. To better understand the differences in annotation quality we have analyzed the individual genes in the 5 genomes listed in Figure 10 further. To enable comparison of annotations, we generated a sequence-based matching of genes between the manually curated version of each of the five genomes (maintained within the SEED) and the corresponding RAST annotated version.

### Detailed discussion of the range of "differences" in annotations

Table 1 shows that between 81.7% (*M. jannaschii*) and 94.9% (*Buchnera*) of genes matched between RAST and SEED have identical annotations.

**Table 1:** Differences in annotation

**Genome**	**genes**	**% matched**	**% identical**	**different**
*Alcanivorax borkumensis *SK2	2814	92.8	93.7	164
*Aquifex aeolicus *VF5	1613	91.7	87.5	185
*Buchnera aphidicola str*. Bp	550	90.0	94.9	25
*Methanocaldococcus jannaschii *DSM 2661	1844	90.6	81.7	306
*Wolinella succinogenes DSM *1740	2094	93.8	84.0	314

The total number of genes (genes) is the number annotated in SEED, percentage of matched genes (% matched) is the number generated by a sequence-based matching of genes. Of those matched genes the % identical subsets are annotated with an identical annotation. The last column gives the number of predicted genes with different annotations.

For the three genomes in Table 2 we have performed a careful manual analysis of the discrepancies in annotation, attempting to reconcile annotations that were not automatically recognized as identical.

**Table 2:** Analysis of the discrepancies in annotation between SEED and RAST for three genomes

**Genome**	**different**	**really different**	**manually reconciled**
*Alcanivorax borkumensis *SK2	164	111	53
*Methanocaldococcus jannaschii *DSM 2661	306	153	153
*Wolinella succinogenes *DSM 1740	314	159	155

As shown in Table 2 a significant percentage of the differing annotations can be manually reconciled. For 4.2% of the 2814 features in *A. borkumensis *SK2 the RAST server did not predict an identical function. Slightly worse results were found for *Methanocaldococcus jannaschii *DSM 2661 (9.1%) and *Wolinella succinogenes *DSM1740 (8.01%). As an example of annotations that were judged as "essentially identical" in our manual comparison, but viewed as distinct by our automated comparison please consider the following pairs:

• Type 4 prepilin-like proteins leader peptide processing enzyme/TYPE IV PREPILIN PEPTIDASE

• phage DNA polymerase domain protein/DNA polymerase, bacteriophage-type

### Detailed discussion of the range of "differences" in gene calls

A number of genes were missed in the RAST predictions of genes present in the contigs, and in addition the RAST server predicted genes that were not present in the manually curated SEED genomes. Table 3 details the results of a careful manual analysis of those differences in gene prediction for three genomes from Table 1.

**Table 3:** A detailed manual analysis

		**missing in RAST**		**missing in SEED**	
**Genome**	**RNAs**	**hypoth.**	**non-hypo**	**hypoth.**	**non-hypo**

*Alcanivorax borkumensis *SK2	51	113	38	49	16
*Methanocaldococcus jannaschii *DSM 2661	43	105	25	74	19
*Wolinella succinogenes *DSM 1740	45	40	44	98	22

A detailed manual analysis of the genes called in RAST and in the SEED sheds some light on the differences in the respective predictions. As the matching was performed on protein sequences, RNAs could not be matched. Genes found in the SEED and not predicted in the RAST were split into two categories with and without hypothetical annotations. Additional predictions found in RAST but not in SEED were also included and again split into hypothetical and non-hypothetical.

The majority of genes missing in RAST or predicted in RAST, but not predicted in SEED, are hypothetical and short.

Our manual analysis of the features predicted by the RAST server shows that only 1.3% (*A. borkumensis *SK2 and *Methanocaldococcus jannaschii *DSM 2661) of the non-hypothetical genes in the SEED and only 2.1% for *Wolinella succinogenes *DSM 1740 were missed by RAST.

Further analysis revealed that in the case of *Methanocaldococcus jannaschii *DSM 2661 of the 44 non-hypothetical genes that the RAST server did not predict, 15 were transposases or recombinases, 5 were small ribosomal proteins and one was a leader peptide. These 21 cases present hard cases for the current gene prediction algorithm used in RAST.

### RAST to KAAS annotation comparison

We have compiled a comparison of RAST annotations to annotations obtained from KAAS for five genomes (*Bacillus subtilis subsp. subtilis *str. 168, *Escherichia coli *K12, *Staphylococcus aureus subsp. aureus *COL, *Synechocystis sp*. PCC 6803, *Vibrio cholera cholerae *O1 biovar eltor str. N16961).  The RAST annotation to KAAS annotation comparison is available at the URL given in the "Availability and requirements section". The KAAS provides functional annotation of genes by BLAST comparisons against the manually curated KEGG GENES database. RAST annotations were obtained by submitting the DNA sequences of the genomes (GenBank format obtained from RefSeq) to the RAST server. KAAS annotations were obtained by submitting the protein sequences for each genome (obtained from RefSeq) to the KAAS. The resulting RAST and KO (KEGG Orthology) assignments have been tabulated for each genome and sorted by GenBank identifiers. In addition each GenBank identifier has been connected to the appropriate entry in the Annotation Clearing House (ACH) [23] to allow comparison to other public annotation resources. The ACH is a framework for comparing annotations of identical proteins from public resources such as: TIGR-CMR [24], UniProtKB/Swissprot and UniProtKB/TrEMBL[12], GenBank [25], SEED [9], DOE-JGI IMG [26], Integrated Microbial Genomes [26], KEGG [27].

### Summary and Discussion of results

There are obvious limitations in using existing SEED genomes to evaluate the service. However we believe that the examples discussed above indicate that the RAST server has a false-negative rate of false gene predictions between 1.3% and 2.1%. While a more comprehensive analysis is possible, the lack of a gold standard for gene predictions in diverse genomes leads the authors to believe that this performance analysis is adequate.

As shown in the examples discussed above the rate of false positives is of the same order or magnitude as the rate of false-negatives (Table 3).

The functional annotations generated by the RAST server are between 91% and 94% identical to those in the SEED. Again the lack of a "gold standard" for annotations makes a more formal evaluation problematic, but we believe that our analysis provides a qualitative estimate of the actual server performance.

The reader is encouraged to manually peruse the comparison of    annotations described in section headed "RAST to KAAS annotation    comparison"  (see also the URL provided in "Availability and requirements section)  to gain an appreciation of the relative accuracies provided by the    different annotation services or to select a well-annotated existing    prokaryotic genome from any source, submit the contigs to the RAST    server, and do a comparison of the returned annotations against those    in the original version.   It is the most direct way to gain a meaningful estimate of accuracy, consistency and completeness.

### Developments in progress

We envision many additions and improvements to the RAST Server several of which are already being addressed by our team and will be discussed in the following paragraphs.

### Detection and Processing of "Foreign DNA"

In many genomes, careful analysis of prophages, the remnants of transposition events, insertions resulting from conjugation, and the resulting pseudogenes is considered an essential part of a manual annotation effort. It will become increasingly important that we provide this analysis rapidly, accurately, and automatically if we wish to process (for example) hundreds or thousands of closely related pathogen genomes.

### Processing Lower-quality Sequence

As we move to an era in which hundreds of genomes of less-than-perfect quality are produced, bioinformatics support will be needed to compensate for frameshifts that reflect errors in sequence data. At this point most annotation efforts are understandably reluctant to alter the input sequence or to derive adjusted protein translations in order to eliminate the impact of what might (or might not) be a frameshift. We will offer a service that allows a user to request automated "correction" of what appear to be frameshifts, recording the alterations in attached annotations.

### A Server that Will Support Analysis of Short Fragments of DNA

A simple modification to the step in which the RAST server establishes the closest phylogenetic neighbours can be used to allow processing of relatively short fragments of DNA (typically over 20 kb). We have added this capability, although it will not be part of this initial release. While the quality of the annotations is undoubtedly inferior to what can be done with complete genomes, we feel that many users would value even the limited analysis we can provide automatically, allowing such a fragment to be explored in a framework designed to support comparative analysis.

### Construction of Analogous RAST-based Servers for Metagenomic Data

We have constructed an analogous server, the MG-RAST (MetaGenome-RAST), that is designed to take as input an environmental sample in the form of thousands of "reads". The server uses many aspects of the technology described within this paper, but also features numerous additions designed to support the analysis of metagenomic data [28].

### Processing More Types of Genes

There is a growing awareness of the need to process more types of RNA genes, as well as properly annotating specialized regions of the genome (e.g., the origin of replication). In many cases, this can be achieved using the growing number of excellent freely available tools that are being developed worldwide. We will certainly add these to the initial step of RAST in which non-protein-encoding genes are recognized before initiating the main analysis.

## Conclusion

We have designed, implemented and released a freely available public server that will provide initial gene calls, gene functions, and metabolic reconstructions for bacterial and archaeal genomes. This server provides initial annotations that we believe to be unusually complete, consistent and accurate. It achieves these goals by utilizing the growing collection of *subsystems *produced by "The Project to Annotate 1000 Genomes" and a collection of protein families, which are referred to as *FIGfams*. The existing implementation is capable of sustaining a throughput rate of 50–100 genomes daily.

## Availability and requirements

The server is freely available at .

The RAST annotation to KAAS annotation comparison is available at 

## Authors' contributions

RKA Subsystem creation and maintenance; DB Evaluation of the RAST output and QC; AAB Development and maintenance of metabolic scenarios; MDeJ Development and maintenance of metabolic scenarios; TD Development and implementation of Rapid Propagation Technology; RAE Subsystem creation and maintenance; KF Development and maintenance of metabolic scenarios; SG Subsystem creation and maintenance; EG Contributed to development of user interface; MK Subsystem creation and maintenance; FM RAST System architecture and evaluation of the output, manuscript preparation; GJO Subsystem creation and maintenance; RO Development and implementation of Rapid Propagation Technology; ALO Subsystem creation and maintenance; RAO RAST System architecture and Development, implementation of Rapid Propagation Technology, manuscript preparation, corresponding author; LKMcN Testing and evaluation of the RAST output; DP Interface design and implementation; TP Interface design and implementation; BP Development and implementation of Rapid Propagation Technology; GDP Development and implementation of Rapid Propagation Technology; CR Testing and evaluation of the RAST output; RS RAST System architecture; OV Subsystem creation and maintenance; VV Subsystem creation and maintenance, manuscript preparation; AW Testing and Monitoring of the RAST server; OZ Subsystem creation and maintenance. All authors have read and approved the final manuscript.
